# Cardiovascular response to short-term fasting in menstrual phases in young women: an observational study

**DOI:** 10.1186/s12905-015-0224-z

**Published:** 2015-08-28

**Authors:** Kumiko Ohara, Yoshimitsu Okita, Katsuyasu Kouda, Tomoki Mase, Chiemi Miyawaki, Harunobu Nakamura

**Affiliations:** Graduate School of Human Development and Environment, Kobe University, Kobe, Japan; Research Fellow of the Japan Society for the Promotion of Science, Tokyo, Japan; Graduate School of Science and Technology, Shizuoka University, Hamamatsu, Japan; Department of Public Health, Kinki University Faculty of Medicine, Osaka-Sayama, Japan; Department of Childhood Education, Kyoto Seibo College, Kyoto, Japan; Department of Early Childhood Education, Heian Jogakuin (St. Agnes’) College, Kyoto, Japan

**Keywords:** Menstrual cycle, Women's health, Dietary bihavior, Fasting, Anti-stress

## Abstract

**Background:**

Menstrual cycle-related symptoms are an important health issue for many women, and some may affect cardiac autonomic regulation. In the present study, we evaluated the cardiovascular and physiological stress response to 12-h short-term fasting in the menstrual phases of healthy young women.

**Methods:**

We performed a randomized crossover study. Subjects were seven female university students (age: 22.3 ± 1.0 years). The experiments comprised four sessions: meal intake in the follicular phase, meal intake in the luteal phase, fasting in the follicular phase, and fasting in the luteal phase. All subjects participated in a total of four experimental sessions during two successive phases (follicular and luteal phase in the same menstrual cycle, or luteal phase and follicular phase in the next menstrual cycle) according to a randomized crossover design. R-R intervals were continuously recorded before and after meals, and power spectral analysis of heart rate variability was performed. Other physiological data were obtained before and 20, 40, 60, and 80 min after meal intake or after the corresponding time point of meal intake (fasting in the follicular or luteal phase).

**Results:**

Heart rate decreased during fasting in the follicular and luteal phases. High frequency power increased during fasting in the follicular and luteal phases. In addition, salivary cortisol concentrations decreased during fasting in the luteal phase.

**Conclusions:**

In the present study, short-term fasting resulted in higher parasympathetic activity and lower cortisol levels in the luteal phase in these young women. These results indicate a possibility to produce an anti-stress effect in the luteal phase, which may reduce menstrual symptoms.

## Background

Menstrual cycle-related symptoms may include psychological symptoms, such as irritation or depressed feelings, or physical symptoms, such as headache or stomach ache in the premenstrual [1–3] and menstrual phases of the menstrual cycle [4, 5]. According to the estimation of World Health Organization (WHO), 199 million women had premenstrual syndrome as of 2010, which was 5.8 % of all women [6]. Previous reports also showed about 10 % of women had premenstrual syndrome, and about 70–90 % of women experienced menstrual cycle-related symptoms [7–13]. This also applies to Japanese women [14–16]. In addition to physiological characteristics, menstrual cycle-related symptoms affect health-related quality of life [17]. According to an epidemiological study, average scores of students with premenstrual syndrome were significantly lower in all domains of quality of life [18, 19]. Therefore, alleviation of these symptoms is expected to contribute to improvement of quality of life.

Previous studies showed that symptoms are severe during the luteal phase [11, 20, 21]. The venous oxygenation index is decreased in the late luteal phase in premenstrual syndrome subjects [22]. In addition, stressful situations have been reported to be associated with the occurrence of menstrual cycle-related symptoms [23]. Stress is known to affect cardiovascular changes, which are usually related to autonomic nervous system activity changes [24]. Spectral analysis of heart rate variability (HRV) provides a sensitive, non-invasive measure of cardiac autonomic regulation. Two main frequency components of HRV have been demonstrated, low frequency (LF: 0.04–0.15 Hz) reflecting both sympathetic and parasympathetic nervous system activity, and high frequency (HF: 0.15–0.40 Hz) reflecting the activity of the parasympathetic nervous system [25]. Consequently, the LF-to-HF (LF/HF) ratio represents the sympathovagal balance [25]. In previous studies, HF was lower in the luteal phase than the follicular phase [26–31], and LF/HF was higher in the luteal phase than the follicular phase [26–29, 32], indicating increased sympathetic nervous activity in the luteal phase and increased parasympathetic nervous activity in the follicular phase. Moreover, sympathetic nervous activity is stronger in women with severe menstrual symptoms than in those with less severe symptoms [33].

Dietary restriction (DR), also known as calorie restriction, is a restriction of total food from ad libitum feeding. There is some evidence that restricting single micronutrients or organic compounds, such as sodium or caffeine, alleviates menstrual cycle-related symptoms [34, 35]. Pellizzer et al. reported that reduced dietary fat intake increased parasympathetic activity in women [36]. Fasting is one form of DR. The anti-stress effects of fasting were demonstrated in men, whereby short-term fasting (12 h) increased parasympathetic domain activity while meal intake activated sympathetic domain activity [37]. However, there is little evidence of the anti-stress effects of short-term fasting on menstrual cycles. Some studies reported that 2.5- to 3.5-day fasting suppresses the reproductive axis [38–40], which means a fasting period is critical. All of these factors lead us to hypothesize that 12-h short-term fasting may increase parasympathetic activity in the luteal phase.

Therefore, in the present study, we evaluated the cardiac autonomic regulation and physiological stress response to 12-h short-term fasting during each menstrual phase in young women.

## Methods

### Subjects

The subjects were recruited from the local university with no incentives. We enrolled seven healthy female students who satisfied the inclusion and exclusion criteria for entry into the study. The inclusion criteria were female gender and age of 20 to 24 years. The exclusion criteria were past and current smoking, alcoholism, high-performance athletes, medication use, oral contraceptive use, irregularity of menstrual cycle, parity, and body mass index ≥ 25 kg/m^2^. All subjects were carefully informed about the purpose and potential risks of this experiment, and all gave written informed consent to participate in this study. The study protocol was approved by the Human Ethics Committee of the Graduate School of Human Development and Environment, Kobe University.

### Experimental procedure

**Fig. 1:**
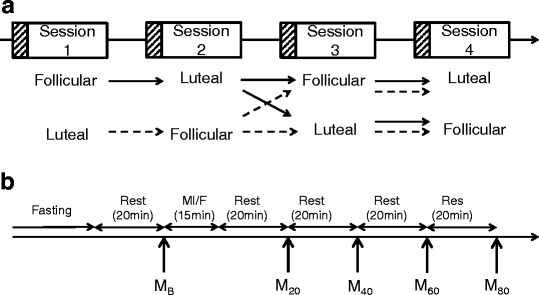
Experiment design. **a** Session and menstrual phase. Subjects were randomly assigned to the follicular session or luteal session. The second session was conducted in the other menstrual phase of the first session. Third and fourth sessions were conducted as well as the first and second session. In the first and second session, the subjects were randomly assigned to the meal intake trial or the fasting trial. In the third and fourth session, the subjects were assigned to the other trial. In each session, baseline and subsequent measurements were conducted. Baseline and skew-patterned square in each session shows 12-h fasting. **b** Experiment procedure in each session. *M*_*B*_ baseline measurement, *M*_*20*_ measurement at 20 min after meal intake, *M*_*40*_ measurement at 40 min after meal intake, *M*_*60*_ measurement at 60 min after meal intake, *M*_*80*_ measurement at 80 min after meal intake, *MI/F* meal intake in the meal intake trial or fasting in the fasting trial

The experiments were conducted from February 2012 to February 2014. The experimental procedure is shown in Fig. 1a and b. All subjects participated in a total of four experimental sessions during two successive phases (follicular and luteal phase in the same menstrual cycle, or luteal phase and follicular phase in the next menstrual cycle) according to a randomized crossover design. In the first menstrual cycle, the first and second sessions were conducted. In the second menstrual cycle, the third and fourth sessions were conducted. In the first and second sessions in the first menstrual cycle, the subjects were randomly assigned to each trial: (1) the meal intake trial or (2) the fasting trial. After a 12-h overnight fast, subjects assigned to the meal intake trial were served a test meal, and those assigned to the fasting trial continued fasting for a further 2 h (total 14 h). If the first session was conducted in the follicular phase, the second session was conducted in the luteal phase in the same menstrual cycle. If the first session was conducted in the luteal phase, the second session was conducted in the follicular phase in the next menstrual cycle. Third and fourth sessions were also conducted in the same way as the first and second sessions. In the first and second sessions, the subjects were randomly assigned to the meal intake trial or the fasting trial. In the third and fourth session, the subjects were assigned to other than the trial assigned in each follicular or luteal phase in the first and second sessions. Subjects were determined as being in the follicular phase or luteal phase based on the occurrence of menstruation and ovulation. Ovulation was recognized by a surge in luteinizing hormone detected using two self-examination kits for luteinizing hormone (L-check FT; Nipro Co., Ltd, Osaka, Japan and P-check; Mizuho Medy Co., Ltd, Saga, Japan).

On the day before testing, the subjects in both the meal intake trial and the fasting trial finished their meals by 21:00 h and went to sleep before 24:00 h. They abstained from drinks containing caffeine, food containing capsaicin, alcohol, and sports activity for 24 h prior to each testing day. After overnight fasting, each subject arrived at the laboratory between 08:00 h and 08:30 h and rested in the supine position for 20 min in an environmentally controlled room (temperature, 23–26 °C; relative humidity, 50–60 %). After resting, baseline physiological and psychological assessments were conducted. Saliva samples were collected at the same time, and stored at −25 °C for assessment of salivary cortisol levels. The physiological assessments included an electrocardiogram, and measurement of systolic blood pressure (SBP) and diastolic blood pressure (DBP). SBP and DBP were measured by the Riva-Rocci-Korotkoff method using a digital manometer (GP-303S, Parama-Tech Co., Ltd, Fukuoka, Japan). A test meal was served for the meal intake trial at 09:50 h, and the subjects finished eating by 10:05 h. Postprandial physiological and psychological data and a saliva sample were obtained at 20, 40, 60, and 80 min after meal intake. For the fasting trial, a test meal was not served, but physiological and psychological data and a saliva sample were obtained 20, 40, 60, and 80 min after the corresponding time point of intake of food in the meal intake trial.

### Composition of nutrients and energy content in each trial

The meal for the experiment consisted of 81 g of bread made from bread flour and white sugar, 26 g of ham, and 200 g of orange juice, which together contained 395 kcal of energy, 15.5 g of protein, 11.3 g of fat, 57.5 g of carbohydrate (energy %: protein/carbohydrate/fat 15/62/23), and 0.8 g of sodium. The overall energy contained in the meal and the energy proportions of carbohydrate, protein, and fat were determined according to the mean intake of food in females in their 20s reported in the National Nutrient Survey in Japan [41]. These meals were served at 36.0 °C. Meal temperature was measured using a non-contact thermometer (Thermo-Hunter, model PT-2LD; Optex Co., Otsu, Japan). All drinks were provided at room temperature. In the fasting trial, each subject continued fasting throughout the experiment for a further 2 h.

### Heart rate variability

The R-R intervals, the time interval between two consecutive R waves in the electrocardiogram, were continuously recorded before and after meals by an ambulatory electrocardiograph monitor (Active Tracer AC-301A; GMS Inc., Tokyo, Japan) with a sampling rate of 1 kHz. Power spectral analysis of HRV was performed using a 16th-order autoregressive model [42] by Kubios HRV analysis software 2.0 (Biomedical Signal and Medical Imaging Analysis Group, Department of Applied Physics, University of Kuopio, Finland) [43].

We analyzed low frequency power (LF: 0.04–0.15 Hz) and high frequency power (HF: 0.15–0.40 Hz) as HRV parameters. LF power reflects sympathetic and parasympathetic modulation of the heart, whereas HF power primarily reflects parasympathetic modulation of the heart [25]. LF and HF power were computed for each minute of the 5-min R-R data [44]. The LF/HF ratio was calculated as an index of sympathovagal balance [25]. The mean of each 1-min HRV was used for statistical analysis.

### Salivary cortisol assay

All frozen samples were thawed, mixed, and centrifuged at 3000 × g for 10 min at room temperature. Samples were analyzed in duplicate with a competitive immunoassay specifically validated for the quantitative measurement of salivary cortisol (Salimetrics LLC, State College, PA, USA). The calibration range was 0.012–3.0 μg/dL.

### Statistical analysis

Data are shown as means ± standard error. The Student’s *t* test was used to detect differences between the trials at baseline. A two-way repeated-measures analysis of variance (ANOVA) was used to investigate the effects of meal conditions (eating or fasting), the effects of time, and interaction effects between condition and time on physiological parameters. The Bonferroni test was used for *post-hoc* analysis. Effect sizes are reported as generalized eta squared (*η*_*G*_^*2*^), which allows direct comparison of within- and between-subjects designs [45], and interpreted according to Cohen’s recommendation of .02 for a small effect, .13 for a medium effect, and .26 for a large effect [46]. Statistical analysis was performed using SPSS Statistics 19 (IBM Inc., Tokyo, Japan). A *P* value less than 0.05 was considered statistically significant.

## Results

### Baseline data

The mean age ± standard deviation of subjects was 22.3 ± 1.0 years. The subjects’ height, body weight, and body mass index were 158.0 ± 3.8 cm, 50.7 ± 4.6 kg, and 20.5 ± 2.1 kg/m^2^, respectively. All subjects completed a meal in approximately 15 min in each session. Table 1 shows baseline data for physiological characteristics. There was no difference in the baseline data.

**Table 1:** Physiological characteristics at baseline in each trial (*n* = 7)

	Follicular phase		Luteal phase	
	Fasting trial	Eating trial	Fasting trial	Eating trial
HR (beats/min)	58.1 ± 8.1	56.3 ± 6.5	59.7 ± 9.2	60.9 ± 12.8
SBP (mmHg)	98.7 ± 13.9	100.4 ± 10.6	97.6 ± 9.4	101.4 ± 8.3
DBP (mmHg)	59.9 ± 9.1	62.5 ± 8.2	60.1 ± 7.4	60.1 ± 6.2

*HR* heart rate, *SBP* systolic blood pressure, *DBP* diastolic blood pressure

Data are mean ± standard deviation

There was no difference in baseline characteristics

### Physiological responses

**Fig. 2:**
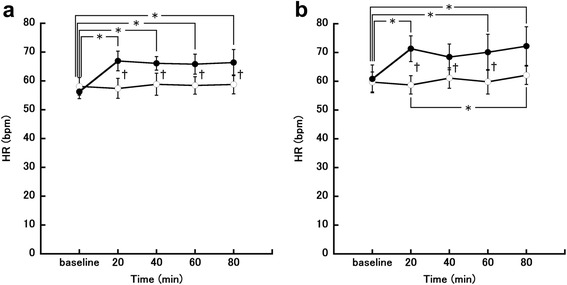
Changes in heart rate (HR). **a** Follicular phase. **b** Luteal phase. The eating trial is shown by closed circles (●; *n* = 7) and the fasting trial is shown by open circles (○;*n* = 7). ^*^ *P* < 0.05, comparison between time points in the eating or fasting session; ^†^ *P* < 0.05, comparison between the eating and fasting trials

Changes in heart rate (HR) in the follicular phase are shown in Fig. 2a. Two-way repeated-measures ANOVA showed that time and meal condition had a significant main effect on HR (time, *P* < 0.001, *η*_*G*_^*2*^ *=* 0.060; meal, *P* < 0.001, *η*_*G*_^*2*^ = 0.118). There was also a significant interaction effect between time and meal condition for HR (F [4, 24] = 6.34, *P* < 0.001, *η*_*G*_^*2*^ *=* 0.056). Post-hoc testing showed that HR in the eating trial was significantly increased at 20, 40, 60, and 80 min after the meal compared with baseline (20 min, *P* = 0.026; 40 min, *P* = 0.004; 60 min, *P* = 0.017; 80 min, *P* = 0.045). There was no significant change in HR in the fasting trial. In addition, HR was significantly higher in the eating trial than in the fasting trial at 20, 40, 60, and 80 min after the meal (20 min, *P* = 0.005; 40 min, *P* = 0.029; 60 min, *P* < 0.001; 80 min, *P* = 0.005). Changes in HR in the luteal phase are shown in Fig. 2b. Two-way repeated-measures ANOVA showed that time and meal condition had a significant main effect on HR (time, *P* = 0.002, *η*_*G*_^*2*^ *=* 0.039; meal, *P* = 0.012, *η*_*G*_^*2*^ *=* 0.120). There was also a significant interaction effect between time and meal condition with HR (F [4, 24] = 5.79, *P* = 0.002, *η*_*G*_^*2*^ *=* 0.030). Post-hoc testing showed that HR in the eating trial was significantly increased at 20, 60, and 80 min after the meal compared with baseline (20 min, *P* = 0.014; 60 min, *P* = 0.037; 80 min, *P* = 0.034). HR in the fasting trial was significantly higher at 80 min after the meal compared with 20 min after the meal (*P* = 0.029). In addition, HR was significantly higher in the eating trial than in the fasting trial at 20, 40, and 60 min after the meal (20 min, *P* = 0.001; 40 min, *P* = 0.009; 60 min, *P* = 0.025).

**Fig. 3:**
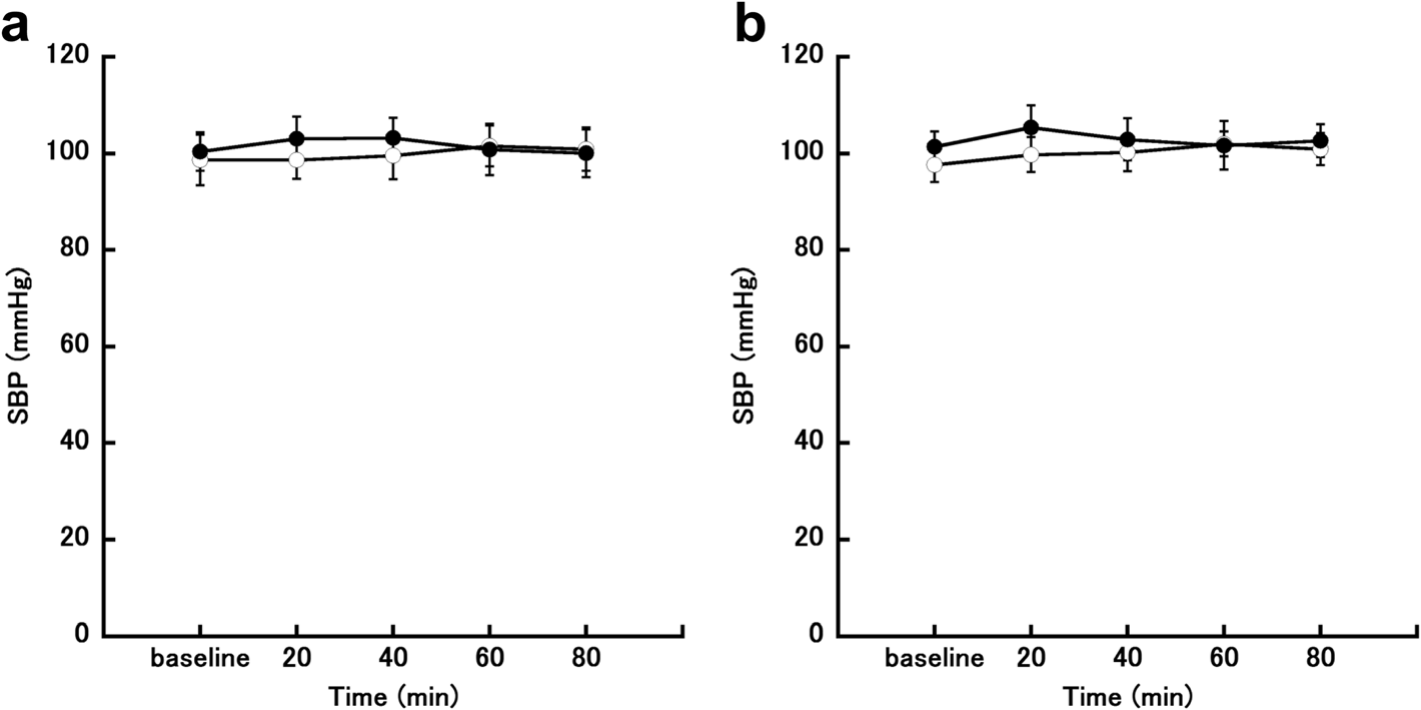
Changes in systolic blood pressure (SBP). **a** Follicular phase. **b** Luteal phase. The eating trial is shown by closed circles (●; *n* = 7) and the fasting trial is shown by open circles (○; *n* = 7)

Changes in SBP in the follicular and luteal phases are shown in Fig. 3a and b. There was no significant main effect or interaction effect for SBP.

**Fig. 4:**
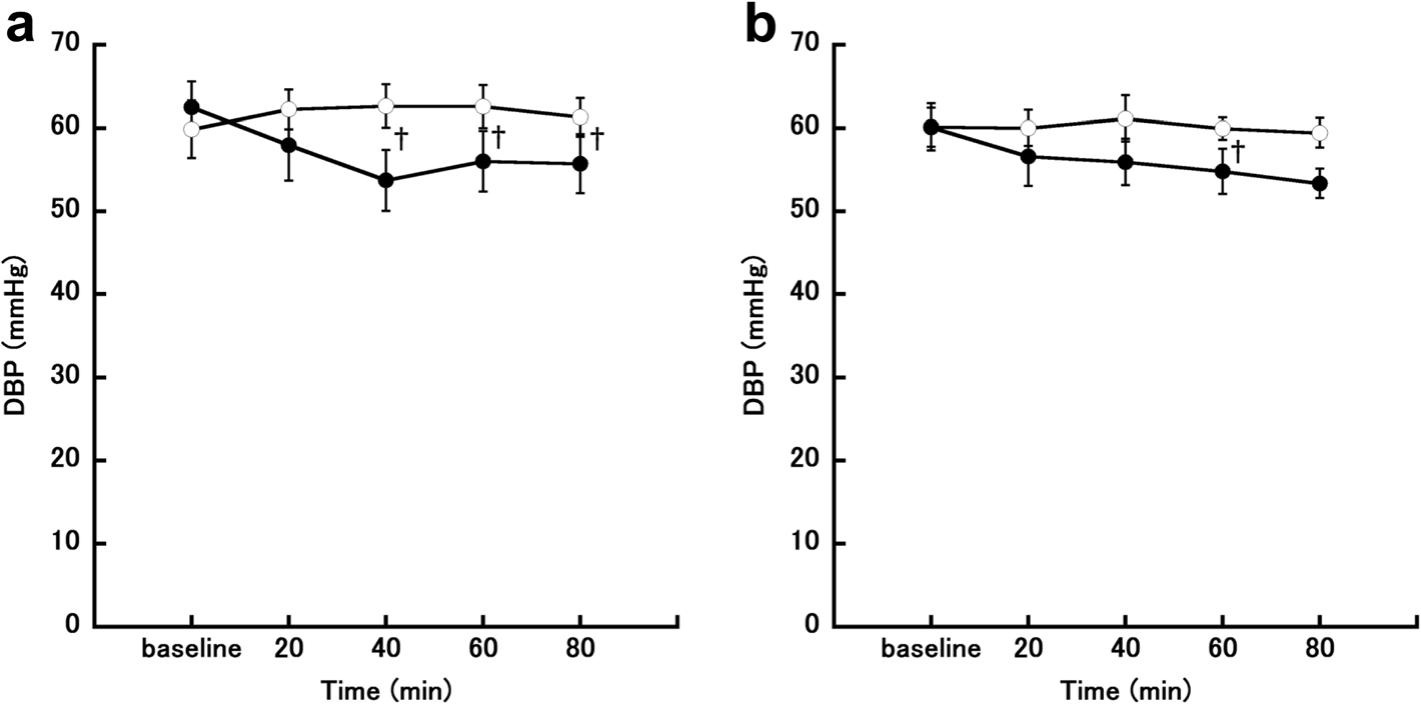
Changes in diastolic blood pressure (DBP). **a** Follicular phase. **b** Luteal phase. The eating trial is shown by closed circles (●; *n* = 7) and the fasting trial is shown by open circles (○; *n* = 7). ^†^ *P* < 0.05, comparison between the eating and fasting trials

Changes in DBP in the follicular phase are shown in Fig. 4a. Two-way repeated-measures ANOVA showed that time had a significant main effect on DBP (*P* = 0.03, *η*_*G*_^*2*^ *=* 0.019). There was also a significant interaction effect between time and meal condition for DBP (F [4, 24] = 5.48, *P* = 0.003, *η*_*G*_^*2*^ *=* 0.058). Post-hoc testing showed that DBP was significantly higher in the fasting trial than in the eating trial at 40, 60, and 80 min after the meal (40 min, *P* = 0.014; 60 min, *P* = 0.049; 80 min, *P* = 0.03). Changes in DBP in the luteal phase are shown in Fig. 4b. Two-way repeated-measures ANOVA showed that time had a significant main effect on DBP (*P* = 0.037, *η*_*G*_^*2*^ *=* 0.040). There was no significant interaction effect between time and meal condition for DBP. Post-hoc testing showed that DBP was significantly higher in the fasting trial than in the eating trial at 40 min after the meal.

### HRV

**Fig. 5:**
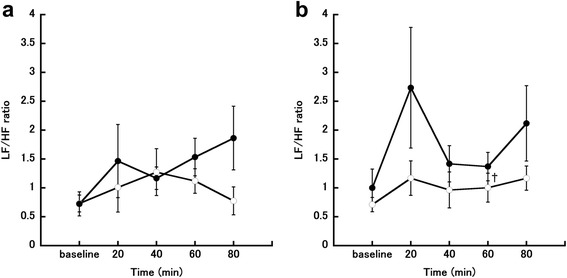
Changes in low frequency/high frequency ratio (LF/HF). **a** Follicular phase. **b** Luteal phase. The eating trial is shown by closed circles (●; *n* = 7) and the fasting trial is shown by open circles (○; *n* = 7). ^†^ *P* < 0.05, comparison between the eating and fasting trials

Changes in the LF/HF ratio in the follicular and luteal phases are shown in Fig. 5a and b. Two-way repeated-measures ANOVA showed that meal condition had a significant main effect on the LF/HF ratio in the luteal phase (*P* = 0.038, *η*_*G*_^*2*^ *=* 0.095). Post-hoc testing showed that the LF/HF ratio was significantly higher in the eating trial than in the fasting trial at 60 min after the meal (*P* = 0.015). There were no other main or interaction effects for the LF/HF ratio.

**Fig. 6:**
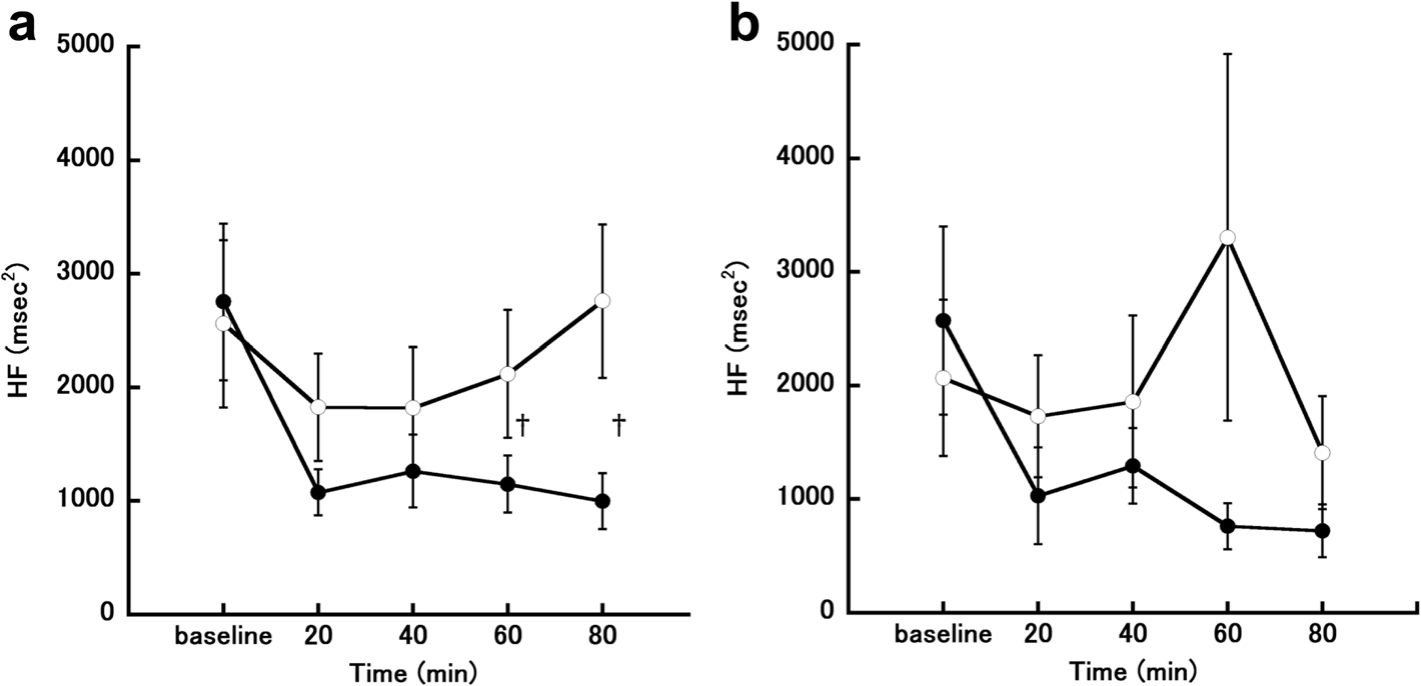
Changes in high frequency (HF). **a** Follicular phase. **b** Luteal phase. The eating trial is shown by closed circles (●; *n* = 7) and the fasting trial is shown by open circles (○; *n* = 7). ^†^ *P* < 0.05, comparison between the eating and fasting trials

Changes in HF power in the follicular phase are shown in Fig. 6a. Two-way repeated-measures ANOVA showed that meal condition had a significant main effect on HF power in the follicular phase (*P* = 0.047, *η*_*G*_^*2*^ *=* 0.088). There was also a significant interaction effect between time and meal condition for HF (F [4, 24] = 2.83, *P* = 0.047, *η*_*G*_^*2*^ *=* 0.061). Post-hoc testing showed that HF power was significantly higher in the fasting trial than in the eating trial at 60 and 80 min after the meal (60 min, *P* = 0.038; 80 min, *P* = 0.018). Changes in HF power in the luteal phase are shown in Fig. 6b. Two-way repeated-measures ANOVA showed that time had a significant main effect on HF power in the luteal phase (*P* = 0.042, *η*_*G*_^*2*^ *=* 0.060). There was also a significant interaction effect between time and meal condition for HF (F [4, 24] = 3.09, *P* = 0.035, *η*_*G*_^*2*^ *=* 0.071).

### Salivary cortisol

**Fig. 7:**
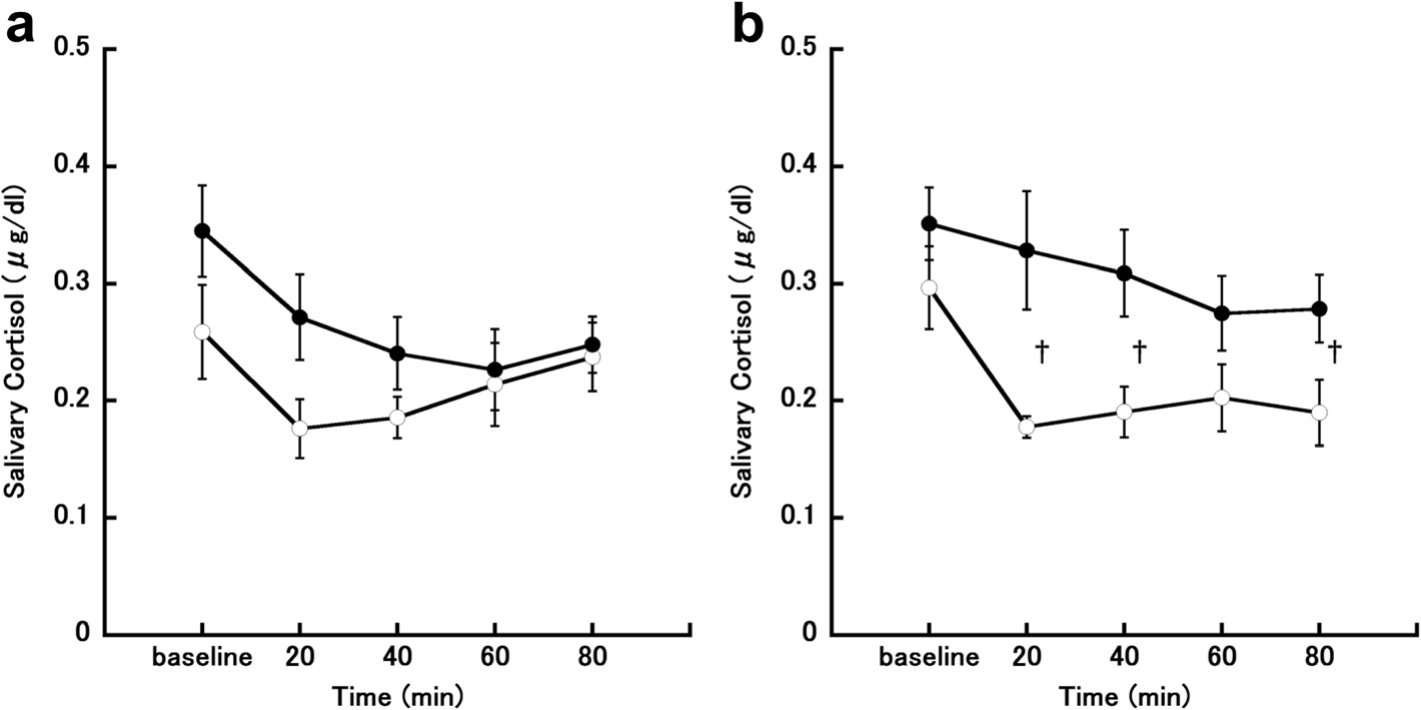
Changes in salivary cortisol concentrations. **a** Follicular phase. **b** Luteal phase. The eating trial is shown by closed circles (●; *n* = 7) and the fasting trial is shown by open circles (○; *n* = 7). ^†^ *P* < 0.05, comparison between the eating and fasting trials

Changes in salivary cortisol concentrations in the follicular phase are shown in Fig. 7a. Two-way repeated-measures ANOVA showed that time had a significant main effect on salivary cortisol concentrations in the follicular phase (*P* = 0.003, *η*_*G*_^*2*^ *=* 0.065). There were no other main or interaction effects in salivary cortisol concentrations. Changes in salivary cortisol concentrations in the luteal phase are shown in Fig. 7b. Two-way repeated-measures ANOVA showed that time and meal condition had a significant main effect on salivary cortisol concentrations (time, *P* = 0.015, *η*_*G*_^*2*^ *=* 0.176; meal, *P* = 0.028, *η*_*G*_^*2*^ *=* 0.319). There was also a significant interaction effect between time and meal condition for salivary cortisol concentrations (F [4, 24] = 3.45, *P* = 0.023, *η*_*G*_^*2*^ *=* 0.066). Post-hoc testing showed that salivary cortisol concentrations were significantly higher in the eating trial than in the fasting trial at 20, 40, and 80 min after the meal (20 min, *P* = 0.011; 40 min, *P* = 0.011; 80 min, *P* = 0.037).

## Discussion

In the present study, we evaluated the cardiovascular response to the short-term fasting in the different menstrual phases. The main results were as follows: (1) HR was decreased during fasting in the follicular and luteal phases; (2) HF power was increased during fasting in the follicular and luteal phases; and (3) salivary cortisol concentrations were decreased during fasting in the luteal phase.

In the present study, during fasting, HR was decreased and HF power was increased in the follicular and luteal phases. Generally, HF power indicates parasympathetic activity [25]. In addition, HR increases in the sympathetic dominant phase and decreases in the parasympathetic dominant phase [47]. Therefore, the present results in the fasting state indicated that parasympathetic activity was dominant. In male subjects, 12-h fasting results in dominance of parasympathetic activity compared with food intake [37]. Conversely, little is known about the effects of short-term fasting such as 12-h fasting in women. Herbert et al. reported that 24 h of fasting induced higher heart rate, and lower HF, indicating increased sympathetic nervous system activity [48]. In addition, 72-h fasting increased norepinephrine, dopamine, and heart rate, and decreased cardiac vagal modulation [49]. These results are inconsistent with our results. The reason for this discrepancy is unclear, but it may be a result of the difference in fasting duration.

In our study, interestingly, salivary cortisol concentrations were decreased during fasting in the luteal phase. Salivary cortisol is reflected by stressful situations. Cortisol concentrations increase during stressful situations and decrease during low-stress situations [50]. The use of a potent laboratory stress protocol reproducing a job interview (e.g., the Trier Social Stress Test [51]) activates the sympathetic–adrenomedullary system and the hypothalamic–pituitary–adrenocortical axis [52]. Both systems interact in managing the adaptive response to stressful events, and biomarkers of these systems can be evaluated non-invasively in saliva. This method allows repeated samples in a short time [53–55]. Activation of the hypothalamic–pituitary–adrenocortical axis induces the secretion of cortisol, which stimulates mobilization of the energy required to overcome the stressor. Therefore, cortisol is considered as the main biomarker in stress research [50]. Based on these facts, the present results indicate that fasting leads to less stressful conditions. These results are consistent with the present results of HR or autonomic activity. Reduction of stress has also been shown in previous reports in some experiments in rats or rhesus monkeys [56, 57] and men [37], but not in women. Interestingly, in the present study, anti-stress effects were shown in female subjects in the luteal phase. This suggests that DR has an anti-stress effect on the luteal phase. Menstrual cycle-related symptoms are severe during the luteal phase [58], and stress is related to these symptoms [23]. Therefore, DR is also expected to reduce these symptoms and increase quality of life in women.

### Limitations

There are some limitations to this study. First, the meals used in the present study had a complicated nutrient content, whereas fasting was a simple experiment. Therefore, the underlying mechanism for the present results is still unclear. The autonomic nervous system engages in maintenance of homeostasis by cooperation with other regulatory systems. Therefore, the underlying mechanism in this study may be explained from the viewpoint of whole-body coordination in future studies. Second, we conducted experiments with a controlled setting, which may be different from a non-controlled setting. Third, we did not investigate the long-term effects of fasting. Long-term effects of food intake are ultimately necessary to clarify the influence of daily lifestyles in humans. Therefore, further studies are required to determine this issue. Fourth, the present study does not refer to longer-term stress. Future studies need to test repeated 12-h fasting to verify the effects of fasting on long-term stress. Repeated 12-h fasting may not only affect long-term stress but also address the feasibility and safety of fasting, and the influence of resumed eating on stress levels.

## Conclusions

We evaluated the cardiovascular response to short-term fasting in each menstrual phase in healthy young females. HR is decreased during fasting in the follicular and luteal phases. HF power is increased during fasting in the follicular and luteal phases. Additionally, salivary cortisol concentrations are decreased during fasting in the luteal phase. These results suggest a possibility of short-term fasting to produce anti-stress effects in the luteal phase in women, which may lead to reduced menstrual symptoms. Twelve-hour fasting is an easy method to practice in daily life without help from a health professional. To that end, further study needs to clarify the mechanisms underlying these findings and to verify the long-term effects of fasting.
